# Eco-friendly fabrication of silver nanoparticles from *Echinops* species: a comparative study of antibacterial and photocatalytic performance

**DOI:** 10.1039/d5ra08508j

**Published:** 2026-02-05

**Authors:** Hafiz Ammar Bin Saeed, Noreen Sajjad, Zarfishan Zulfiqar, Zain Fatima, Muhammad Ajaz Hussain, Gulzar Muhammad, Abid Ali, Amel Y. Ahmed, Maryam Kaleem

**Affiliations:** a Department of Chemistry, Institute of Chemical Sciences, Government College University Lahore 54000 Lahore Pakistan mgulzar@gcu.edu.pk; b Department of Chemistry, The University of Lahore 1-Km Defence Road Lahore Pakistan noreensajjad1623@gmail.com kaleemmaryam569@gmail.com; c Centre for Organic Chemistry, School of Chemistry, University of the Punjab Lahore 54590 Pakistan; d Department of Chemistry, Faculty of Science, King Faisal University Al Ahsa 31982 Saudi Arabia aebrahim@kfu.edu.sa

## Abstract

The green synthesis of metal nanoparticles (NPs) has been of growing interest, in part because it is environmentally friendly, less toxic, and uses plant-derived phytochemicals as natural reducing and stabilizing agents, providing a more sustainable approach to traditional chemical synthesis. This study reports the green synthesis of silver NPs (Ag NPs) from aqueous leaf extracts of *Echinops ritro* and *Echinops spinosus* and assesses the comparative antibacterial and photocatalytic properties. The optical band gap energies of Ag NPs grown using both plants were determined to be 2.76 eV and 2.78 eV, respectively. FTIR, SEM, and XRD analyses have identified the functional groups in the formation of polydisperse NPs and validated their size and crystalline structure. The synthesized Ag NPs-ES demonstrated the best antibacterial activity with a maximum inhibition zone (24.66 mm) against *S. aureus*. In comparison, the zone of inhibition (ZOI) against other strains was 24 ± 1, 21.66 ± 0.88, and 21 ± 0.57 mm for *B. licheniformis*, *B. Subtilis*, and *E. coli*, respectively, while Ag NPs-ES showed the same trend in the maximum ZOI against *S. aureus* (22.33 ± 0.33 mm), followed by *B. subtilis* (20.66 ± 0.66 mm), *B. licheniformis* (15.33 ± 0.88 mm), and *E. coli* (15 ± 0.57 mm). The photocatalytic degradation of methylene blue (MB) and methyl orange (MO) dyes under sunlight was more prominent with Ag NPs from *E. spinosus* (80% & 88%) than from *E. ritro* (71.2% & 74.8%), following pseudo-first-order kinetics with higher rate constants. The results supported that *E. ritro* and *E. spinosus*-capped Ag NPs are potent, environmentally friendly materials with potential applications in antibacterial formulations and wastewater treatment.

## Abstract 2

An ecofriendly green synthesis of silver nanoparticles using *Echinops* species is presented, with a comparative evaluation of their antibacterial efficacy and photocatalytic degradation performance.

## Graphical abstract

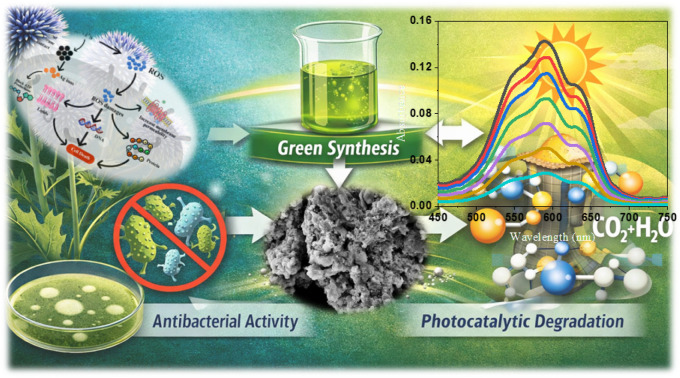

## 1. Introduction

The field of nanotechnology demonstrates rapid advancement through developments that enable the production of new materials at the nanoscale.^1^ Biosynthesis of NPs represents an eco-friendly, safer, and more economical method compared to chemical and physical synthesis methods.^2,3^ Plant extracts demonstrate excellent potential for green NP synthesis because they are widely available, cost-effective, and easily scalable.^4–6^ The bioactive molecules contained in these extracts comprise polyphenols,^7^ along with reducing sugars, nitrogenous bases, and amino acids, that help reduce metal ions in precursor solutions.^8^ Nucleation follows the initial reduction of metal ions, and therefore, extra interrelated ions are generated to stabilize NP growth. Plant extract biomolecules also improve the stability of the extract and prevent aggregation of the particles.^9^ The biosynthesis technique of producing NPs results in decreased toxicity than chemical techniques, making it applicable in various fields.^10^

These particles have a heavy size of nanoscale that leads to the formation of unique physicochemical properties with an increase in surface to volume ratio and altered atomic interaction as compared to bulk-scale materials.^11,12^ NPS classification is based on their make-up, as they are made out of metallic or non-metallic substances.^13^ Ag, gold, copper, cobalt, and nickel are the primary metallic NPs combined with semiconductor materials. The construction components of most non-metallic NPs are mainly carbon-based.^14^ The research community pays much attention to metallic NPs, which are very operational due to their extraordinary electrical characteristics, as well as optical properties and catalytic activity.^15^ Ag NPs are distinguished from metallic NPs because of their outstanding characteristics.^16–18^ Ag NPs were long appreciated in human civilization as a source of medicine and preservation.^19^ Because Ag is a central element, it shows strong antibacterial action when manufactured in the form of NPs, as they have a greater surface area for contact with microbes.^20^

Scientists use Ag NPs in solar energy, spectrally selective coatings, and battery technology for intercalation purposes and chemical reactions as catalysts.^21^ Accepted biomedical applications of Ag NPs include bio-labeling and antibacterial coatings, together with wound healing operations.^22^ The antibacterial attributes of Ag NPs enable their use as protection agents in textile production, together with food and plastic material manufacturing, to prevent microbial growth.^23^ The rising consumer demand for Ag-based nanomaterials prompted scientists to create various fabrication methods for metallic silver and Ag-containing compounds.^24^ Ag NPs serve as eco-friendly antibacterial nano-coatings for paints^25^ and act as antibacterial components in food packaging systems.^26^ Antibacterial properties of Ag ions have made Ag NPs an effective food conservator and additive for various food products.^27^ Ag NPs have been demonstrated to accelerate wound recovery and better cosmetic healing, as well as minimize scar development through animal research.^28^ Ag NP's properties illustrate their increasing importance in scientific and industrial sectors, thereby facilitating future progress in nanotechnology.

Green synthesis of Ag NPs using plant extracts is well documented, but *E. ritro* and *E. spinosus* are not yet fully taken into account in this context. Their phytochemical profiles are different and have not been comparatively investigated regarding their influences on the synthesis and morphology of NPs, as well as their functional activity. Phytochemicals present in *Echinops* species, including various kinds of phytochemicals that can serve as reducing and stabilizing agents in the synthesis of nanoparticles. Phytochemical studies of *E. ritro* and *E. spinosus* have previously reported the presence of phenolic compounds, flavonoids, alkaloids, terpenoids, tannins, and saponins, as well as polysaccharides and reducing sugars. These biomolecules also contain the following subunits: hydroxyls, carbonyls, and amine groups, which have been shown to increase metal-ion reduction and nanoparticle stabilization.^29,30^ The variations in the character and levels of these phytochemicals across *Echinops* species may also influence the nanoparticle nucleation and growth, morphology, and functional activity, including antibacterial and photocatalytic ones.

To date, no systematic comparative study has been found on *E. ritro* and *E. spinosus* with respect to green silver nanoparticle synthesis and dual antibacterial and photocatalytic evaluation. In the article, Ag NPs were synthesized and compared across two species, where their abilities in dye degradation (MB and MO) were tested using photocatalytic reactions, as well as analyzing their antibacterial and antibiofilm activities. The article presents a novel ecologically sustainable way of developing versatile Ag NPs with potential environmental and biomedical applications. Ag NPs in this study were prepared through the green process with the use of leaf extract of two plants, namely *E. ritro* and *E. spinosus*. The change of color of the solution and NP synthesis spectrum by the UV-visible spectrophotometer proved the synthesis of NPs. The functionalities in both extracts and NPs were determined by FTIR spectroscopy. SEM-EDX was used for determining the morphology of Ag NPs. The dyes (MB and MO) were photo-catalytically degraded using the synthesized NPs. The NPs were also assessed for their antibacterial and antibiofilm activity.

## 2. Experimental

### 2.1. Materials

Leaves of *E. ritro* and *E. spinosus* were purchased from the local market, Lahore, Pakistan. Analytical grade MB (>98.97%), MO (>98.97%), and silver nitrate (>99.97%) were used, which were procured from Sigma-Aldrich. Department of Microbiology, Government College University Lahore, provided tested bacterial strains, McFarland turbidity standard, LB nutrient broth, and Mueller–Hinton agar.

### 2.2. Preparation of plant extract

Leaves of both plants were dried, washed, and dried in sunlight. The powdered leaves (500 g) from each plant were added to boiling water and stirred for 1 h. The extract was filtered with nylon cloth and was stored in a cool place. A fresh stock solution was prepared and used for each experiment.

### 2.3. Synthesis of Ag NPs-ES and Ag NPs-ER

**Fig. 1:**
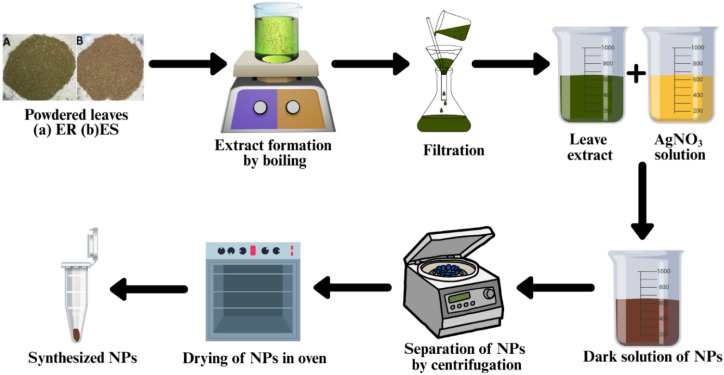
Schematic representation of the synthesis of Ag NPs.

Silver nitrate of different concentrations (20, 40, and 50 mM) was mixed with leaf extracts of both plants in different ratios (1 : 1, 1 : 2, and 2 : 1) at room temperature. The resulting solution, containing 50 mM silver nitrate and a 2 : 1 (extract to salt) ratio, turned dark red after 30 min, indicating the formation of NPs (Fig. 1). The prepared NPs were centrifuged at 2000 rpm for 20 min. After being separated, they were further washed and then dried overnight in an oven.

### 2.4. Characterization

Synthesized NPs were characterized using the UV-visible spectrophotometer. A Shimadzu-1700 Japan UV-visible spectrophotometer provided the absorption spectra in the 200–800 nm wavelength range at room temperature. The absorption spectrum data were used to calculate the band gap energy by Tauc's relationship (eqn (1)).

1 (*αhυ*) = *A*(*hυ* − *E*_g_)^*n*^

Here, the energy-dependent absorption coefficient is denoted as *α*, *E*_g_ represents the band gap energy, *h* is Planck's constant, and *υ* is the frequency. The power factor of transition mode is represented by “*n*,” which is typically 1/2 or 2 for the indirect and direct transition, respectively.

The FTIR (Nicolet iS50 Spectrophotometer) characterized the functional groups present in both leaf extracts and synthesized NPs in the range of 4000 to 400 cm^−1^. The surface morphology and composition of NPs were confirmed by SEM using Nova Nano-SEM 450. At room temperature, the suspension of NPs was dispersed and later dehydrated on SEM grids for determining the morphology of NPs. The crystalline structure and the size of NPs were determined by XRD (Proto AXRD LPR) with copper as the irradiation source. The NPs were scanned at a rate of one step per second at 30 mA current and 30 kV at 2*θ* from 5° to 80°. The sizes were calculated using the Scherrer formula (eqn (2)):2
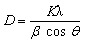
where *D* is the crystallite size, *K* is the shape factor having a value of 0.94, *λ* is the X-ray wavelength (0.15406 nm for Cu Kα), *β* is the full width at half maximum (FWHM) in radians, and *θ* is the Bragg angle.

### 2.5. Antibacterial activity

The Ag NPs were tested for their antibacterial potential against *B. subtilis*, *B. licheniformis*, *E. coli*, and *S. aureus* using the agar well diffusion method. The cell concentration of test pathogens was maintained at 0.5 McFarland turbidity standard. Agar plates were prepared, and wells were made with the help of a cork. The pathogens were spread on a plate with a cotton swab, and sample compounds (50, 100, and 150 µL) were added to the wells. Distilled water and rifampicin were used as negative and positive controls, respectively. The plates were incubated for 24 h at 37 °C, and the ZOI was calculated.

For determining the MIC and MBC values of NPs, 24 h-old bacterial cultures were used. In a sterile test tube, freshly prepared nutrient broth (3 mL) and the bacterial strain (30 µL) were added. Different concentrations of samples (5, 10, 15, 20, 25, 30, 35, 40, 45, and 50 µL mL^−1^) were added to the experimental test tubes, and no compound was added to the control test tubes. The test tubes were incubated at 35 °C for 24 h. After 24 h, the optical density of the test tubes was checked at 523 nm. MICs (100 µL) were spread onto nutrient plates and incubated for 24 h for the determination of MBC.

### 2.6. Photocatalytic degradation

Preliminary optimization experiments were conducted to evaluate the effects of initial dye concentration, pH, catalyst dosage, contact time, and plant extract type. Based on these trials, the optimal conditions were identified and consistently used throughout the study. The efficiency of Ag NPs for the degradation of MB and MO dyes was studied by varying reaction times (20, 40, 60, 80, 100, and 120 min). The Ag NPs (7 mg) were then added to a dye solution (50 mL, 10 ppm) and exposed to sunlight with a high UV index (10+), with the pH maintained at a near-neutral value (6). The solutions were vigorously stirred continuously to ensure the homogenous distribution of dye and NPs. The photocatalytic degradation reactions were undertaken in direct sunlight on clear-sky days at a range of solar irradiance intensity, approximately 80–110 W m^−2^. All experiments were completed at midday (10:00–11:30 am) to avoid any variation in sunlight intensity due to the solar position. Even though standardized UV or visible light sources were not used in this study, natural light (sunlight) was deliberately used to assess the feasibility of the developed Ag NPs in a real environment. Changes in ambient conditions, such as slight fluctuations in sunlight due to passing clouds, were recorded with the samples exposed for the same duration. The MB and MO solution without NPs served as the control group to compare dye degradation and evaluate the effect of sunlight on the dye alone. The samples were scanned with a UV-visible spectrophotometer between 300 and 700 nm wavelengths at different points. Maximum degradation efficiency was calculated under the optimized conditions (eqn (3)).3
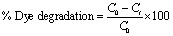
Here, *C*_0_ and *C*_*t*_ are the concentrations of dyes before and after the treatment with NPs. The pseudo-first-order model was used for the kinetics study of dye degradation, and the “*k*” constant was calculated using the equation given below (eqn (4)):4
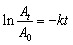
In this equation, *A*_*t*_, *A*_0_, *t*, and *k* are the concentrations at time *t*, concentration at time zero, temperature, and overall rate constant for the dye degradation, respectively.

### 2.7. Statistical analysis

All antibacterial studies were carried out in triplicate (*n* = 3), and the results were presented as mean ± standard deviation. Statistical analysis was conducted to guarantee the reliability and reproducibility of data. Statistical significance was assumed at *p* < 0.05.

## 3. Results and discussion

### 3.1. UV-visible spectroscopy

**Fig. 2:**
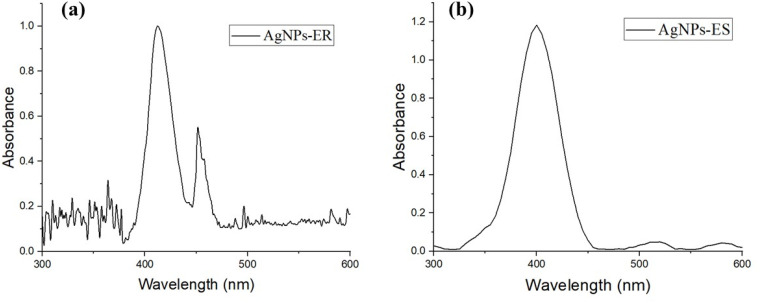
UV-vis spectrum of (a) Ag NPs-ER and (b) Ag NPs-ES.

The Ag NPs characterization for absorption pattern, functional group identification, morphological elucidation, and crystallographic interpretation was done using UV-vis spectroscopy, FTIR, SEM, and XRD. The color change of the reaction mixture provided the first indication of the successful formation of Ag NPs. The solution turned darker within 7–10 min after the plant extract was added to the solution of silver salt (2 : 1). The color shift shows that the reduction of Ag^+^ to Ag^0^ is underway, and the nucleation process has already started, which leads to the creation of NPs.^31^ The synthesis of the Ag NPs was confirmed further by the UV-vis spectral analysis (Fig. 2).

**Fig. 3:**
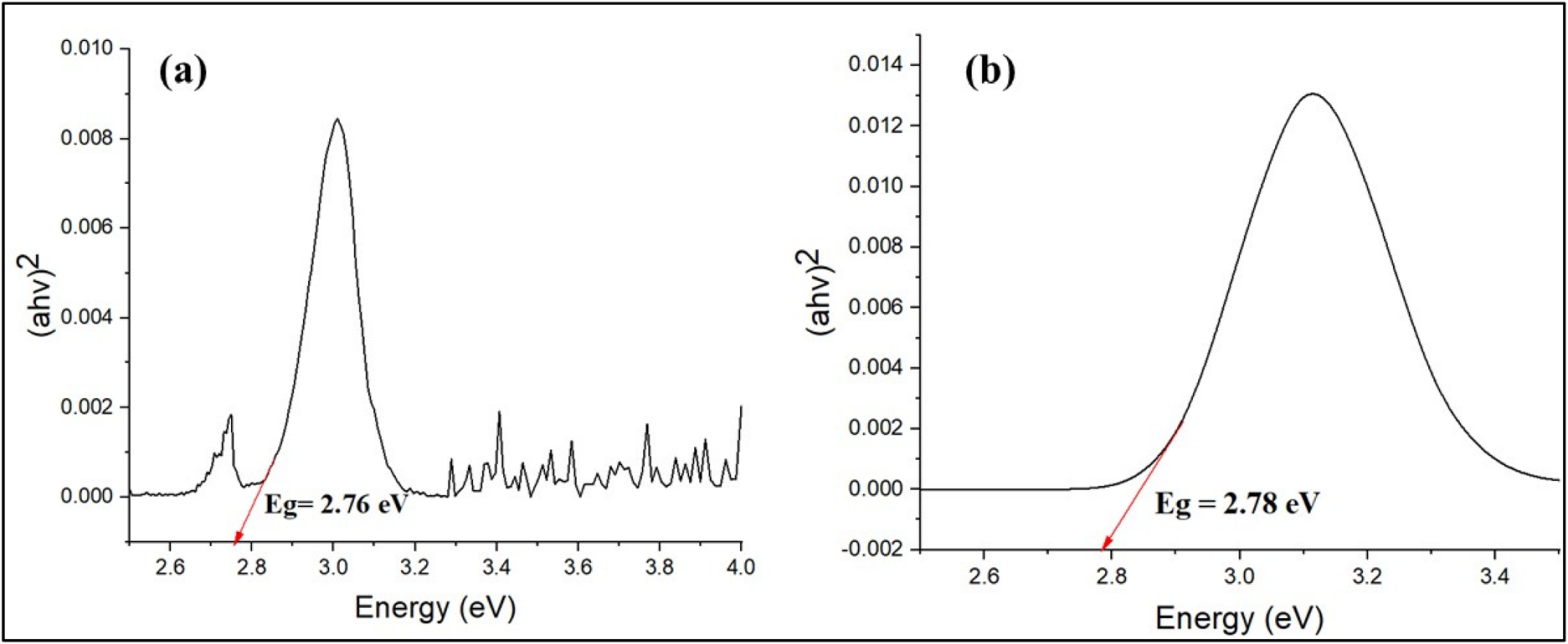
Optical band gap by Tauc plot (a) Ag NPs-ER (b) Ag NPs-ES.

Ag NPs *E. ritro* (Ag NPs-ER) and *E. spinosus* (Ag NPs-ES) gave a surface plasmon resonance band (SPR) at 430 nm and 401 nm, respectively. The irregular shape and agglomeration of the NPs to form a multiple SPR mode is attributed to the shoulder peaks of the UV-vis spectrum of Ag NPs-ER. Conversely, the absence of a shoulder peak in Ag NPs-ES is a sign of a more uniform and symmetrical distribution of particles. These findings prove the effective synthesis since they are in the typical range of Ag NPs provided in the literature.^32^ The SPR differences in the Ag NPs-ER (430 nm) *versus* the Ag NPs-ES (401 nm) are due to the difference in the size, shape, and surface effects due to the difference in the phyto-compounds composition of the extracts of *Echinops*. Ag NPs-ER SPR affected by the red-shift and Ag NPs-ES SPR by the blue-shift correspond to larger particles or partial aggregation, and smaller and more uniformly dispersed nanoparticles with a stronger surface-stabilizing force, respectively. Moreover, the optical band gap of the synthesized NPs was determined by the Tauc plot. As shown in Fig. 3, the band gap calculated was 2.76 eV for Ag NPs-ER and 2.78 eV for Ag NPs-ES, which shows a strong absorption in the visible-light range and semiconducting nature.

### 3.2. FTIR spectroscopy

**Fig. 4:**
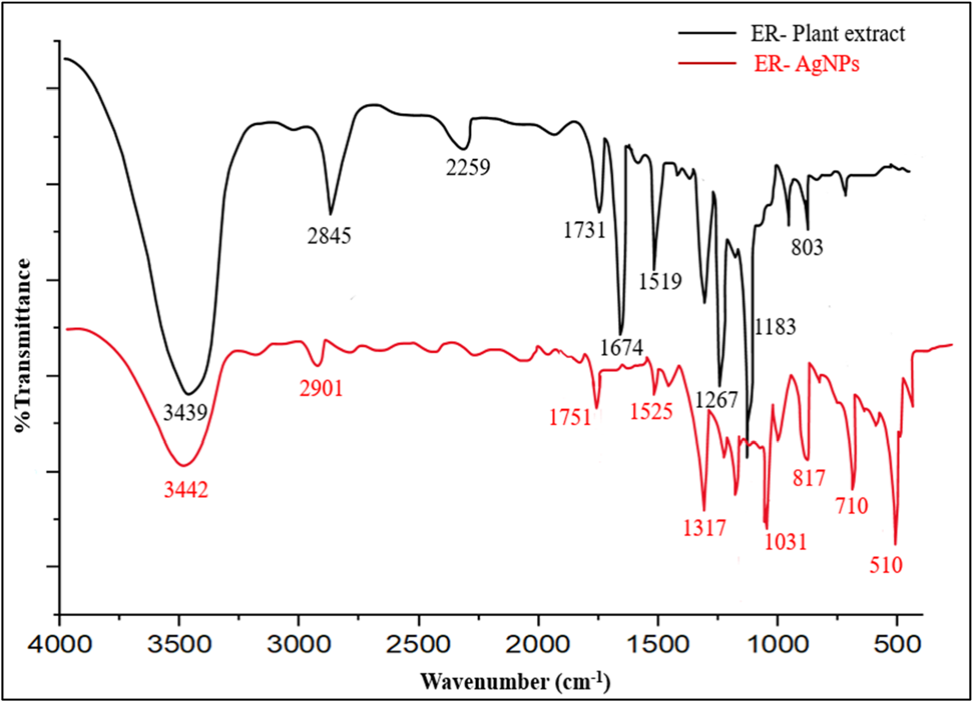
Comparative FTIR spectrum of ER extract and Ag NPs-ER.

**Fig. 5:**
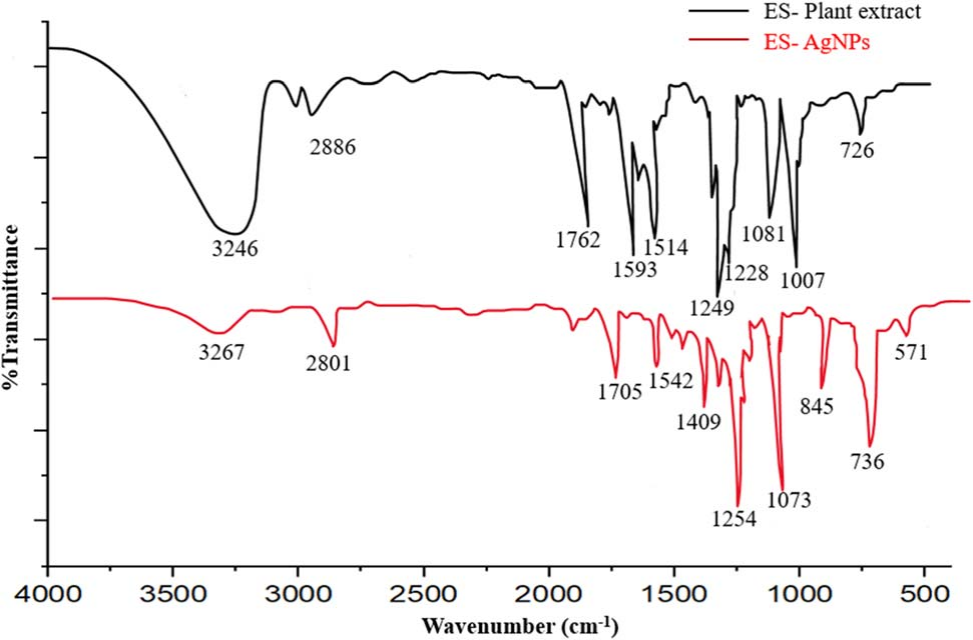
Comparative FTIR spectrum of ES extract and Ag NPs-ES.

FTIR spectroscopy was used to investigate the functionalities in *E. ritro* and *E. spinosus* aqueous extracts and the phytochemicals contributing to the capping of biosynthesized Ag NPs. Comparative FTIR spectra of plant extracts and the respective Ag NPs of both species are presented in Fig. 4 and 5. The FTIR spectrum of the plant extract in the case of *E. ritro* disclosed a broad absorption band at 3439 cm^−1^ corresponding to O–H stretching vibrations owing to hydroxyl groups, commonly occurring in alcohols and phenolic chemicals. 2845 and 2259 cm^−1^ peaks were a result of –C–H stretch vibration of alkanes and stretching of the triple bond (C≡C or C≡N), respectively. Peaks at 1731 and 1674 cm^−1^ are characteristic stretching vibrations of carbonyl (C=O) groups for aldehydes or ketones. The other significant peaks were at 1519 and 1267 cm^−1^, which correspond to nitro and amine functional groups, respectively. The peaks at 1183 and 803 cm^−1^ correspond to C–O stretching and C–H bending, respectively. A significant shift in the position of the peaks and emergence of new bands were noted, confirming the presence of Ag NPs-ER in the FTIR spectrum. In contrast, the peak at 2901 cm^−1^ corresponds to the C–H stretching vibrations. Other peaks were observed at 1751, 1525, 1317, and 1031 cm^−1^, indicating that the carbonyl and amine compounds changed exposure to silver ions. Peaks at 817, 710, and 510 cm^−1^ corresponded to the presence of metal–oxygen or metal–nitrogen (Ag–O/Ag–N) interactions,^33,34^ indicating that plant metabolites were involved in the stabilization of the NPs.

Similarly, FTIR spectrum of *E. spinosus* extract indicated that it had a broad stretching peak of the OH group (3246 cm^−1^) and a peak in CH stretching vibration (2886 cm^−1^). Carbonyl and aromatic C=C stretching was observed to give strong absorption bands of 1762 cm^−1^ and 1593 cm^−1^. The 1514, 1228, 1081, and 1007 cm^−1^ were attributed to nitro compounds, C–N stretching, and C–O–C vibrations, respectively. A distinct peak was observed at 726 cm^−1^, corresponding to C–H out-of-plane bending vibrations. The FTIR spectrum of Ag NPs-ES revealed various significant changes after the formation of NPs. The intensity of the OH band at 3267 cm^−1^ decreased, and the stretching of the CH at 2801 cm^−1^ indicates that the phytochemicals interact with Ag^+^ ions. The involvement of carbonyl, aromatic, and amine groups in the reduction and capping processes was confirmed by the appearance of new and shifted peaks in 1705, 1542, 1409, and 1254 cm^−1^.^35^

Changes in functional-group vibrations and the appearance of new bands in the NPs spectra in both species show the involvement of phenols, flavonoids, alkaloids, and proteins in the bio-reduction of the silver ions and stabilization of the NPs.

### 3.3. SEM analysis

**Fig. 6:**
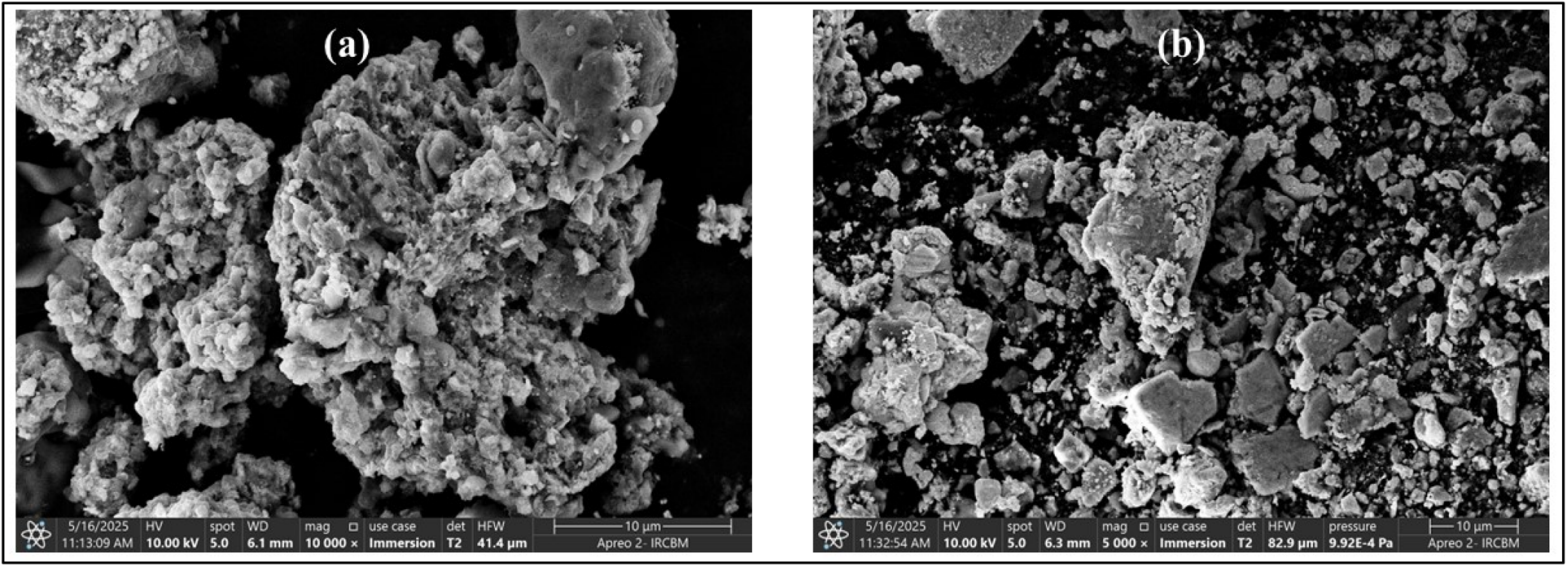
Morphological elucidation through SEM (a) Ag NPs-ER and (b) Ag NPs-ES.

SEM micrographs show irregular, polydisperse particles of moderate size (Fig. 6). The high level of agglomeration in Ag NPs-ER could be due to the low efficiency of the capping and stabilizing phytochemicals present in *E. ritro*. Poor passivation on the surface encourages particle–particle aggregation. Additionally, the broad size distribution indicates that polydispersity may be extensive and can also lead to agglomeration due to variation in surface energy and growth kinetics during NPs synthesis.

In contrast, Ag NPs-ES demonstrated relatively better morphology and lower agglomeration, possibly due to more efficient stabilization by phytochemicals from *E. spinosus*. Morphological data support the confirmation of the successful green synthesis of Ag NPs in their characteristic shape and features on their surface.

### 3.4. XRD analysis

**Fig. 7:**
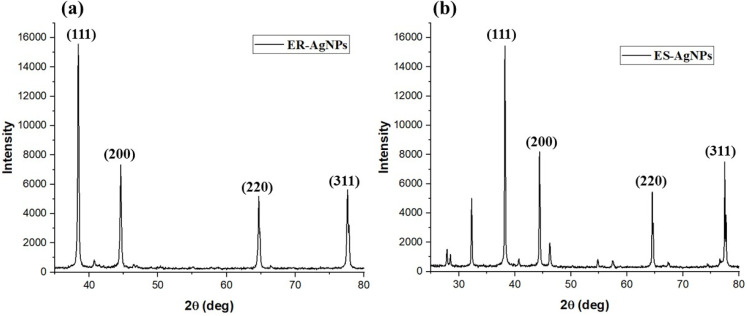
Crystallographic confirmation through XRD (a) Ag NPs-ER and (b) Ag NPs-ES.

The *E. ritro* and *E. spinosus* NPs were characterized by XRD to identify the crystalline structure and phase purity of the synthesized Ag NPs (Fig. 7a and b). According to standard results in JCPDS Card No. 04-0783, the Ag NPs-ER's XRD pattern revealed sharp and distinct peaks at 2*θ* diffraction angles of approximately 38.2, 44.4, 64.5, and 77.5°, which correspond to the (111), (200), (220), and (311) face-centered cubic silver crystallographic planes, respectively. The intense peak corresponding to the 111 plane indicates a preferential orientation and a highly crystalline nature of the synthesized NPs.^35^

Similarly, there were notable diffraction peaks at 38.4, 44.5, 64.7, and 77.6° in the Ag NPs-ES XRD spectrum. These peaks are near the face-centered cubic planes (111), (200), (220), and (311) (JCPDS card no. 04-0783). Furthermore, smaller peaks were observed at lower angles of approximately 27.7 and 32.2, which correspond to the remaining organic compounds or bio-organic capping agents in the plant extract and to crystalline impurities in the naturally occurring biomolecules. However, the bright peaks relating to metallic silver confirm the effective synthesis of crystalline Ag NPs.^36^

The X-ray diffraction (XRD) was used to calculate the average crystallite size of the synthesized silver Ag NPs using the Scherrer equation. Ag NPs prepared with the extract solution (Ag NPs-ES) had an average size of 37.54 nm. On the other hand, Ag NPs-ER showed a smaller average size, with a sample average of 33.5 nm. A Gaussian was fitted to each of the four peaks to estimate FWHM. The FWHM of a fitted Gaussian curve is reported as the FWHM of the peak (Table 1).

**Table 1:** Crystallite size (*D*) of Ag NPs-ES and Ag NPs-ER from XRD data

Sample	2*θ* (degrees)	FWHM (degrees)	Crystallite size (*D* nm^−1^)
Ag NPs-ES	38.22594	0.19728	44.52
	44.41468	0.21905	40.88
	64.55997	0.28996	33.83
	77.50343	0.34411	30.93
Ag NPs-ER	38.40903	0.21899	40.34
	44.58710	0.26027	34.45
	64.70676	0.31322	31.33
	77.64527	0.38250	27.88

The strong and sharp diffraction peaks indicate that highly ordered, tiny (NPs) sized particles were formed. The intensity of the (111) peak in both Ag NPs-ER and Ag NPs-ES is due to the thermodynamic stability and high-density packing of the crystallographic facet of silver, which is a prominent trait observed in Ag NPs synthesized *via* green synthesis routes.^36^ The slight shoulder peaks in the XRD pattern, particularly in Ag NPs-ES, can be attributed to minor crystalline impurities or to weak crystalline organic remnants from the plant extract. These shoulders may also arise from partial overlap of diffraction planes or from the formation of different crystalline domains with slightly different lattice parameters. In addition, such shoulders can indicate lattice strain, defects, or a slight degree of structural disorder introduced during the green synthetic process. The FTIR results are further supported by the crystalline structures observed in both cases, indicating that the phytochemicals present in *E. ritro* and *E. spinosus* convert Ag ions into metallic silver and stabilize the NPs by forming a surface-capping layer, without altering their crystalline order.

### 3.5. Biological and environmental applications

#### 3.5.1. Antibacterial activity

**Fig. 8:**
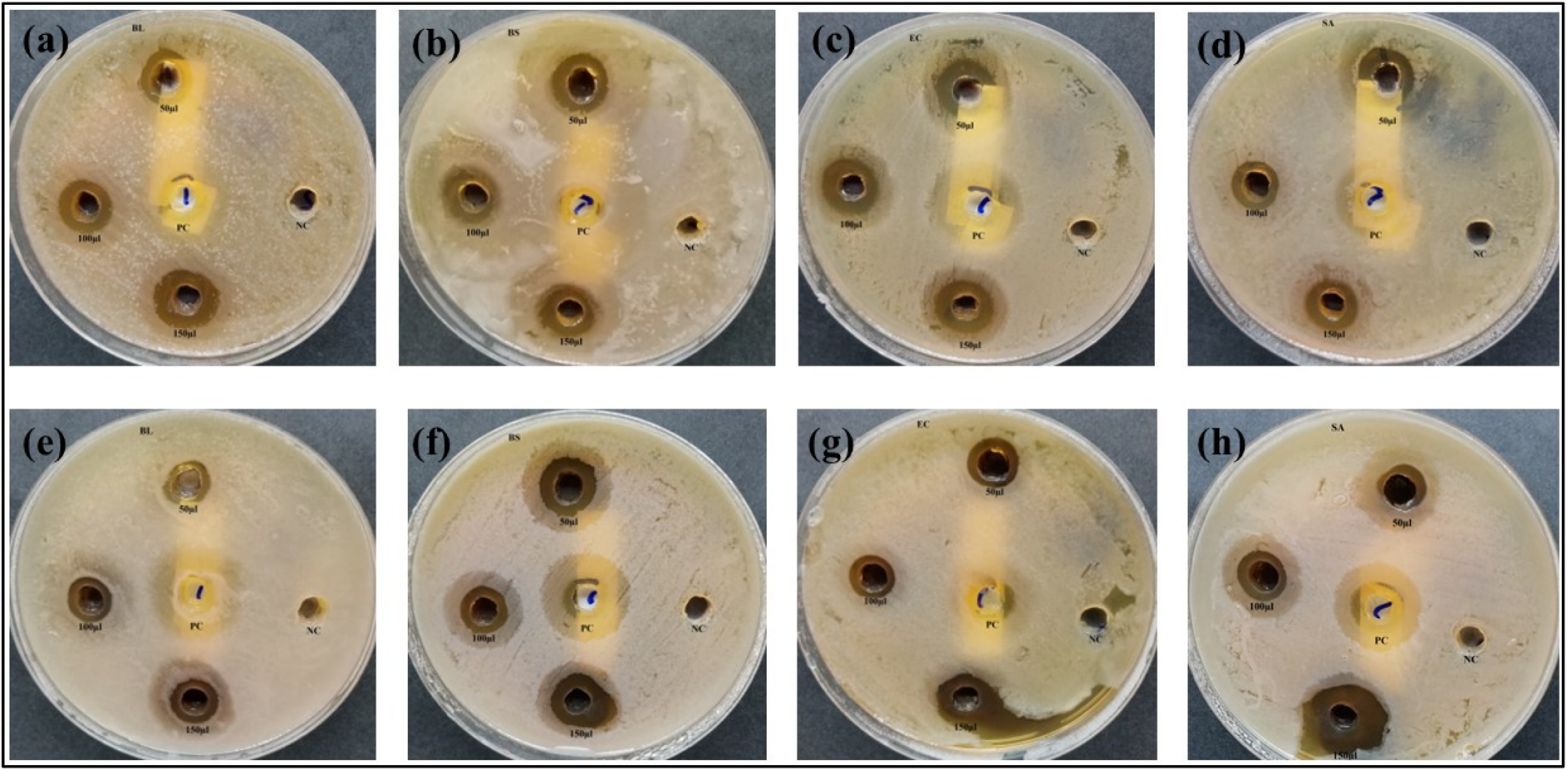
ZOI of 50, 100, and 150 µL Ag NPs-ER, Ag NPs-ES, PC, and NC against (a)–(d) *B. licheniformis*, *B. subtilis*, *E. coli*, and *S. aureus* for Ag NPs-ER, and (e)–(h) the same strains for Ag NPs-ES.

The antibacterial activity of *E. ritro* and *E. spinosus* extracts, as well as their respective Ag NPs (Ag NPs-ER and Ag NPs-ES), was evaluated using the well diffusion method. The evaluation of antibacterial potential focused on measuring the inhibition zone formation in bacterial cultures after subjecting them to different strengths of extracts, ranging from 50 to 150 µL (Fig. 8).

**Fig. 9:**
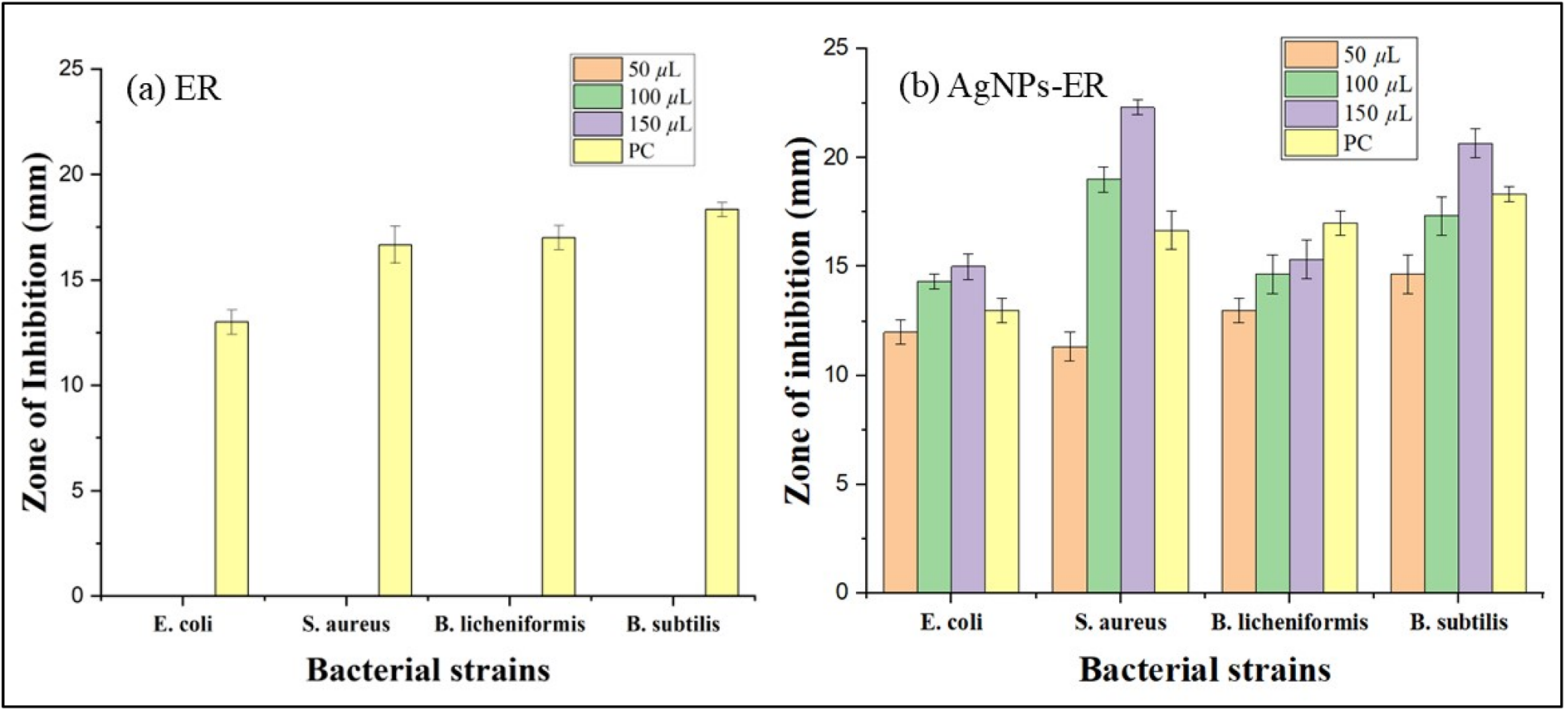
Inhibition zones through bar graphs (a) *E. ritro* extract and (b) Ag NPs-ER against different bacterial strains. Each bar represents mean ± SD.

**Fig. 10:**
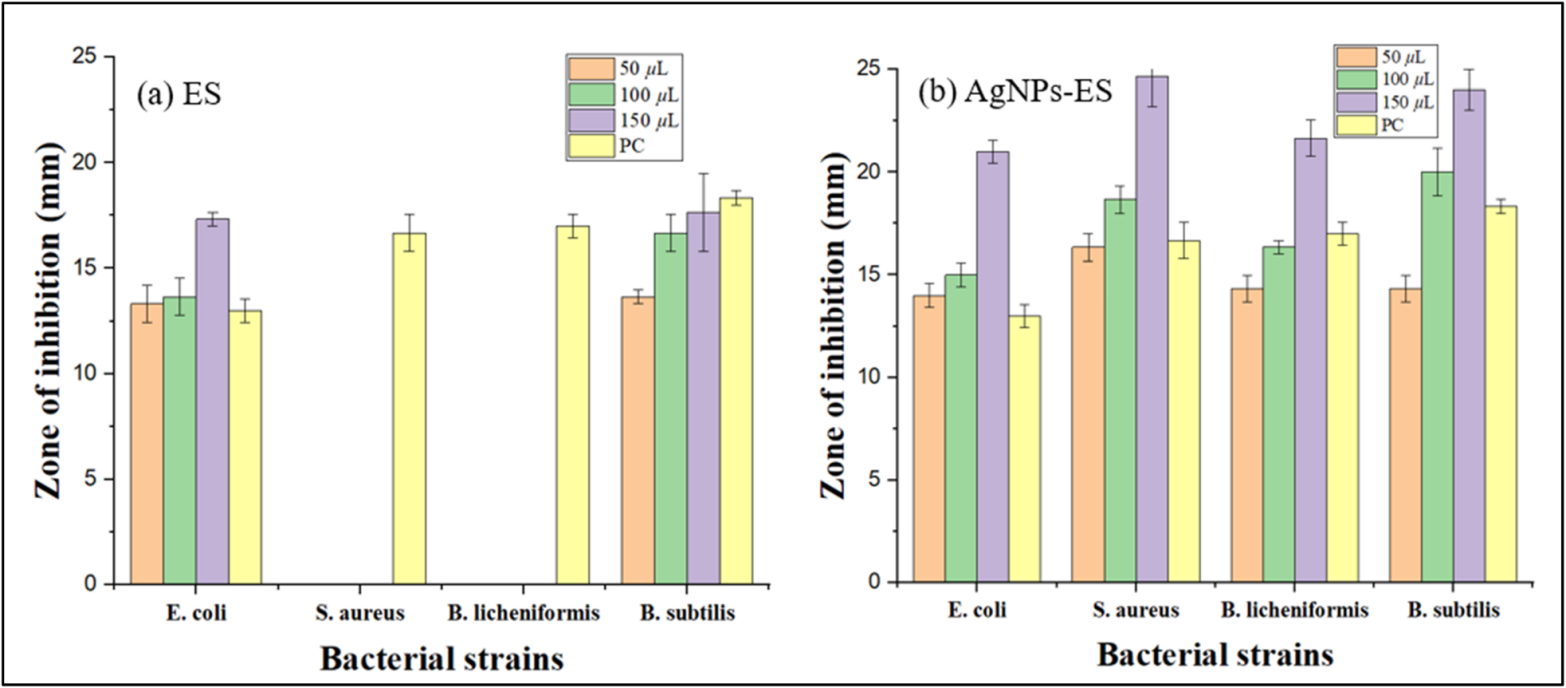
ZOI (mm) against different bacterial strains (a) ES extract and (b) Ag NPs-ES. Each bar represents mean ± SD.

The effectiveness of Ag NPs-ER with respect to antibacterial properties was quite high upon testing against all the tested bacterial strains. A150 µL solution of nanoparticles provided the largest zone of inhibition of 22.33 ± 0.33 mm against *S. aureus*. *B. subtilis*, *B. licheniformis*, and *E. coli* followed with 20.66 ± 0.66 mm, 15.33 ± 0.88 mm, and 15 ± 0.57 mm zones of inhibition, respectively. *B. subtilis* and *S. aureus* exhibited larger antibacterial zones of inhibition than the positive control, indicating that Ag NPs-ER has better antibacterial properties (Fig. 9 and 10). The enhanced antibacterial effect of Ag NPs can probably be explained by their interaction with bacterial cell membranes, leading to cellular damage through the denaturation of proteins and the formation of reactive oxygen species.

Ag NPs-ES exhibited antibacterial effects, which created bigger inhibition zones compared to the positive control against the bacterial strains. *S. aureus* was found to have the largest antibacterial zone with a 150 µL dose, with a size of 24.66 ± 1.45 mm, with *B. subtilis*, *B. licheniformis*, and *E. coli* showing 24 ± 1, 21 ± 0.57, and 21.66 ± 0.88 mm, respectively. A size-dependent effect, as well as shape variations and surface alterations of Ag NPs-ES, is perhaps the reason behind the stronger antibacterial activities in comparison to Ag NPs-ER.

Ag NPs-ES exhibited better antibacterial activity against all bacterial strains as indicated by a larger ZOI when compared to Ag NPs. It is interesting to note that Ag NPs-ES had the greatest ZOI against *S. aureus* (24.66 ± 1.45 mm), making it the most susceptible strain. The Gram-negative *E. coli* exhibited less sensitivity to both Ag NPs-ER and Ag NPs-ES, further confirming the intrinsic resistance characteristic of this bacterium to the outer membrane defense mechanism.

**Fig. 11:**
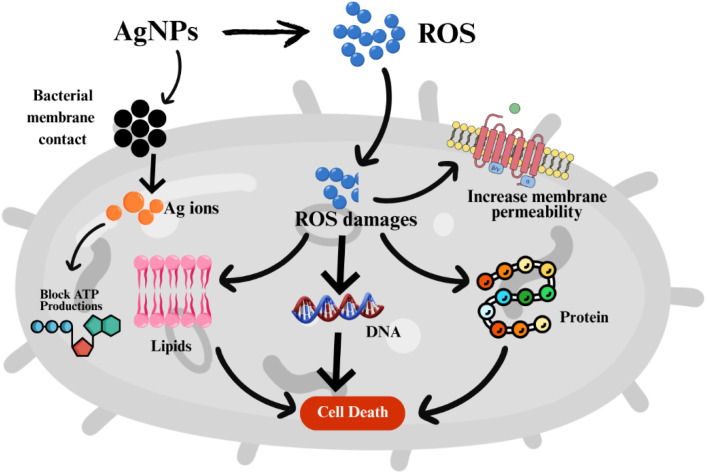
Plausible mechanism of action of antibacterial activity of Ag NPs.

Ag NPs exhibit well-documented antibacterial behavior through various mechanisms, including bacterial membrane contact, ROS generation, and disruption of essential metabolic pathways.^43^ Through cellular contact between Ag NPs and bacterial membranes, membrane permeability is increased, leading to bacterial cell lysis. Ag NPs induce oxidative stress by generating ROS that damage proteins, lipids, and nucleic acids, resulting in bacterial death. The antibacterial effect of Ag NPs results from two active mechanisms: the release of silver ions that block cellular ATP production, as well as the disruption of cellular respiration processes caused by released silver ions, as shown in Fig. 11.^44–46^

**Fig. 12:**
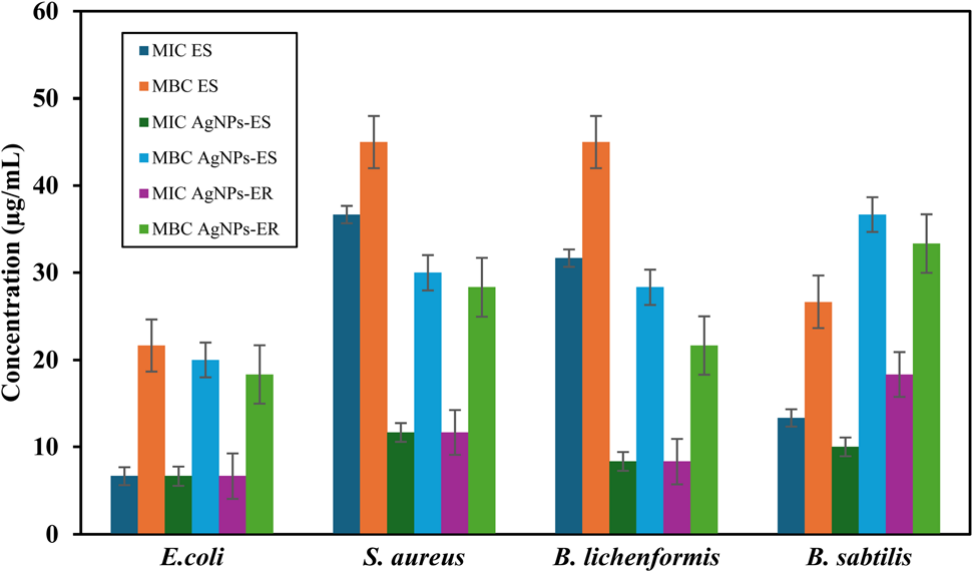
Bar graph representing concentrations as MIC and MBC and their respective Ag NPs. Each bar represents mean ± SD.

The bactericidal and bacteriostatic effect of both plant extracts and their respective NPs was measured. In crude extract, *E. spinosus* depicted moderate activity against *E. coli* and *B. subtilis* (MIC 6.66 and 13.33 µg mL^−1^). Nonetheless, the poorer effects are indicated by higher MIC values against *S. aureus* (36.66 µg mL^−1^) and *B. licheniformis* (31.66 µg mL^−1^). The MBC values ranged from 21.66 to 45 µg mL^−1^. Comparatively, the *E. ritro* (ER) extract exhibited no antibacterial properties, as MIC and MBC values, as well as inhibition and bactericidal concentrations, were not measurable for any of the samples. Ag NPs synthesized using Ag-ER exhibited a notable enhancement in antibacterial activity and the MIC values of 6.66–18.33 µg mL^−1^ against *E. coli*, *S. aureus*, *B. licheniformis*, and *B. subtilis*, respectively. Once again, increased activity of Ag-ES was also noticed, especially on *E. coli* (MIC: 6.66 µg mL^−1^), *B. subtilis* (10 µg mL^−1^), and *B. licheniformis* (8.33 µg mL^−1^) (Fig. 12).

Overall, Ag-ER was superior to all the other specimens tested, considering that Ag-ER inhibitory capacity was high against *B. licheniformis* and *S. aureus*. Ag-ER and Ag-ES had the same potency for *E. coli*. Evidence for these results is the fact that Ag NPs integration improves antibacterial properties considerably in comparison to crude plant extracts.

The results of the research indicated that Gram-negative bacteria (*E. coli*) were more resistant than three Gram-positive strains (*S. aureus*, *B. subtilis*, and *B. licheniformis*) exposed to Ag NPs. Gram-negative bacteria have lipopolysaccharide as the main constituent of the outer membrane that shields the internal contents of the bacteria from exposure to NPs. The bacteria use two defense mechanisms to attack NPs: they are ejected by efflux pumps, and changed into inert materials by periplasmic enzymes.^47^ Gram-positive bacteria lack an outer membrane, thus making them more sensitive to NPs exposure, as the exposure leads to membrane disruption and creates oxidative stress, thus promoting the antibacterial effects.^48^

#### 3.5.2. Dye degradation potential of Ag NPs

Ag NPs prepared using *E. spinosus* and *E. ritro* were evaluated for photocatalytic breakdown analysis of methylene blue (MB) and methylene orange (MO) over 120 min. The degradation was monitored by the UV-vis spectrophotometry, which involved following the reduction in the intensity of absorption at a particular wavelength over time. Without Ag NPs, no changes in the absorbance of either methylene blue (MB) or methyl orange (MO) were detected, indicating that direct photolysis in sunlight did not play a significant role in degrading the dyes. This confirms that the photocatalytic activity of the produced Ag NPs is the main catalyst for the degradation process.

#### 3.5.3. Degradation of methylene blue

**Fig. 13:**
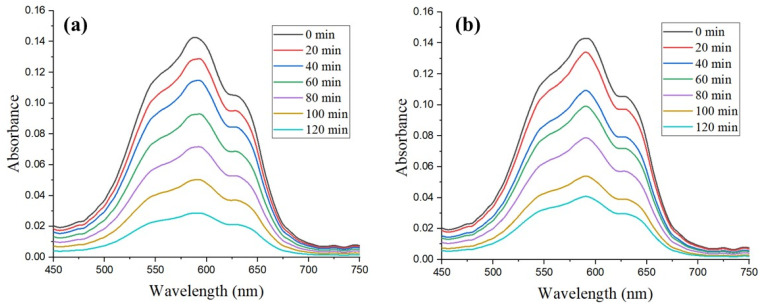
Photocatalytic degradation pattern of MB dye by (a) Ag NPs-ES and (b) Ag NPs-ER.

Fig. 13 demonstrated that the absorbance of MB declined gradually with time when it was exposed to Ag NPs-ES and Ag NPs-ER. The characteristic MB absorption peak at 664 nm slowly decreased as the degradation progressed. The maximum absorbance and reduction were found at 0 and 120 min, respectively, before absorption and reduction declined gradually until 20, 40, 60, 80, 100, and 120 min.

Ag NPs-ES (80%) exhibited greater degradation ability than Ag NPs-ER (71.2%) owing to the greater reduction in absorbance during the experiment. The various catalytic capabilities of Ag NPs may be due to differences in particle size and surface area, and their ROS properties.^49^ The process of degradation takes place because of the plasmonic characteristics of Ag NPs, which elevate the work of photocatalysts and promote transfer of electrons and radical formation, disintegrating MB molecules.^50^

#### 3.5.4. Degradation of methylene orange

**Fig. 14:**
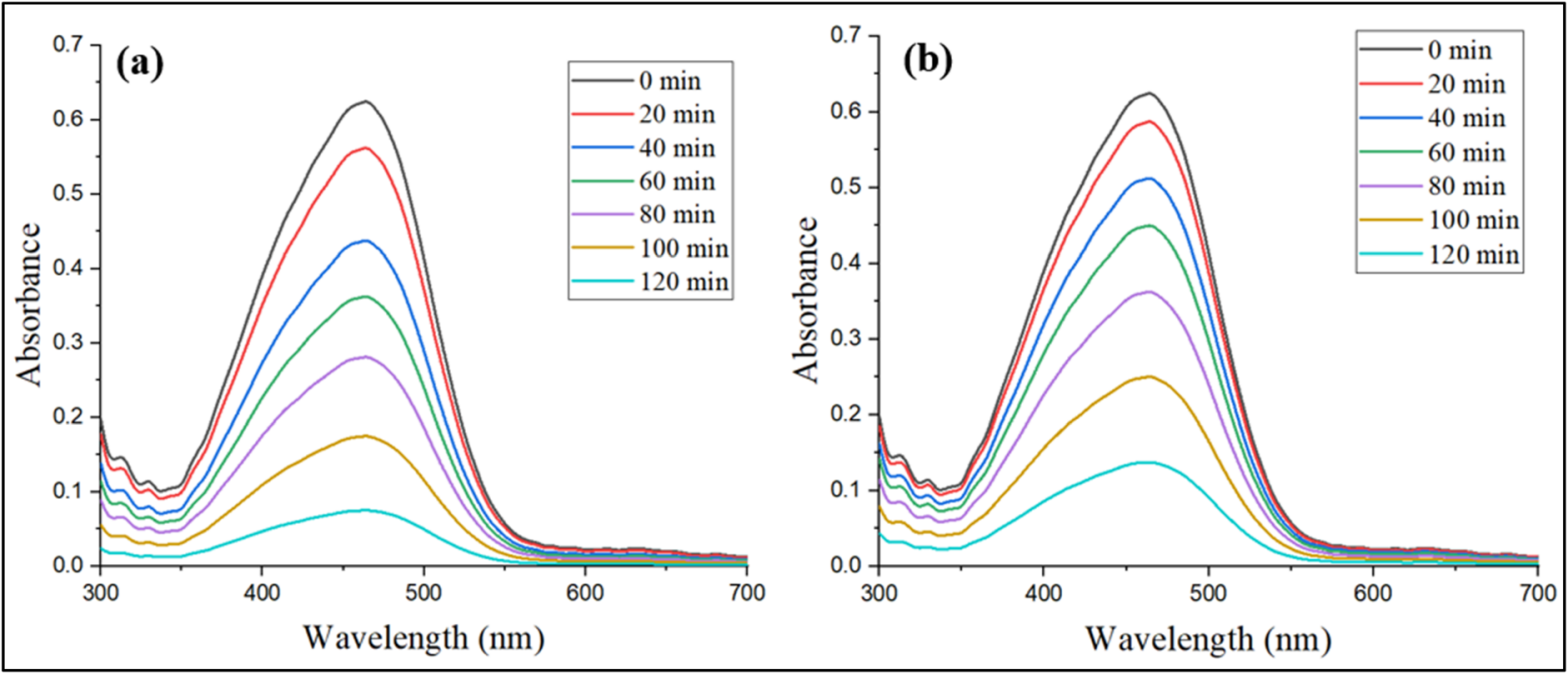
Photocatalytic degradation pattern of MO dye by (a) Ag NPs-ES and (b) Ag NPs-ER.

Fig. 14 shows the degradation pattern of MO using the analysis of UV-vis spectroscopy. The absorption peak of MO is at 464 nm, and the value declined with a reaction time of 120 min. Ag NPs-ES and Ag NPs-ER were effective in degrading MO, where Ag NPs-ES was more effective than Ag NPs-ER, since it degraded the MO by 88% as opposed to 74.8% by Ag NPs-ER.

The MO absorbance pattern shows that Ag NPs allow the quick photocatalytic disintegration of azo chemical bonds present in the dye molecules *via* their photocatalytic effect. When Ag NPs interact with light, they are excited and generate electron–hole pairs and thus begin the degradation process. The use of Ag NPs, water, and oxygen results in the appearance of hydroxyl radicals and superoxide ions, which enhance the degradation of dye.^51^ The enhanced functionality of Ag NPs-ES seems to be connected with their specific morphology of NPs and the encapsulating agents of plant extracts, as these specifics may influence the activity of electrons and the efficiency of the reactions.

The similar patterns of reduction of the MB and MO absorptions indicate the performance of Ag NP as a photocatalyst by showing a reduction in the absorbance with time. The decrease in maximum intensity indicates chromophore destabilization, as Ag NPs trigger oxidative reactions.^52^ Biomolecules obtained from plants and incorporated in the Ag NPs synthesis process enhance the photochemical catalysis by stabilizing NPs and enhancing their proper dispersion. The degradation capacity of Ag NPs derived from *E. spinosus* showed slightly better outcomes than Ag NPs derived from *E. ritro* during both dye degradation processes. The unique biomolecules present in plants appear to influence how NPs form and interact with their environment. The difference in the degradation rates of MO and MB may be due to the variation in their molecular structure. The azo (–N=N–) bonds in MO are more readily oxidatively cleaved by ROS than the heterocyclic structure of MB,^53^ which may not be readily destroyed and might demonstrate relatively lower degradation ability. Also, the surface charge between Ag NPs and dye molecules might affect photocatalytic behavior.^54^

#### 3.5.5. Kinetic studies of dye degradation

**Fig. 15:**
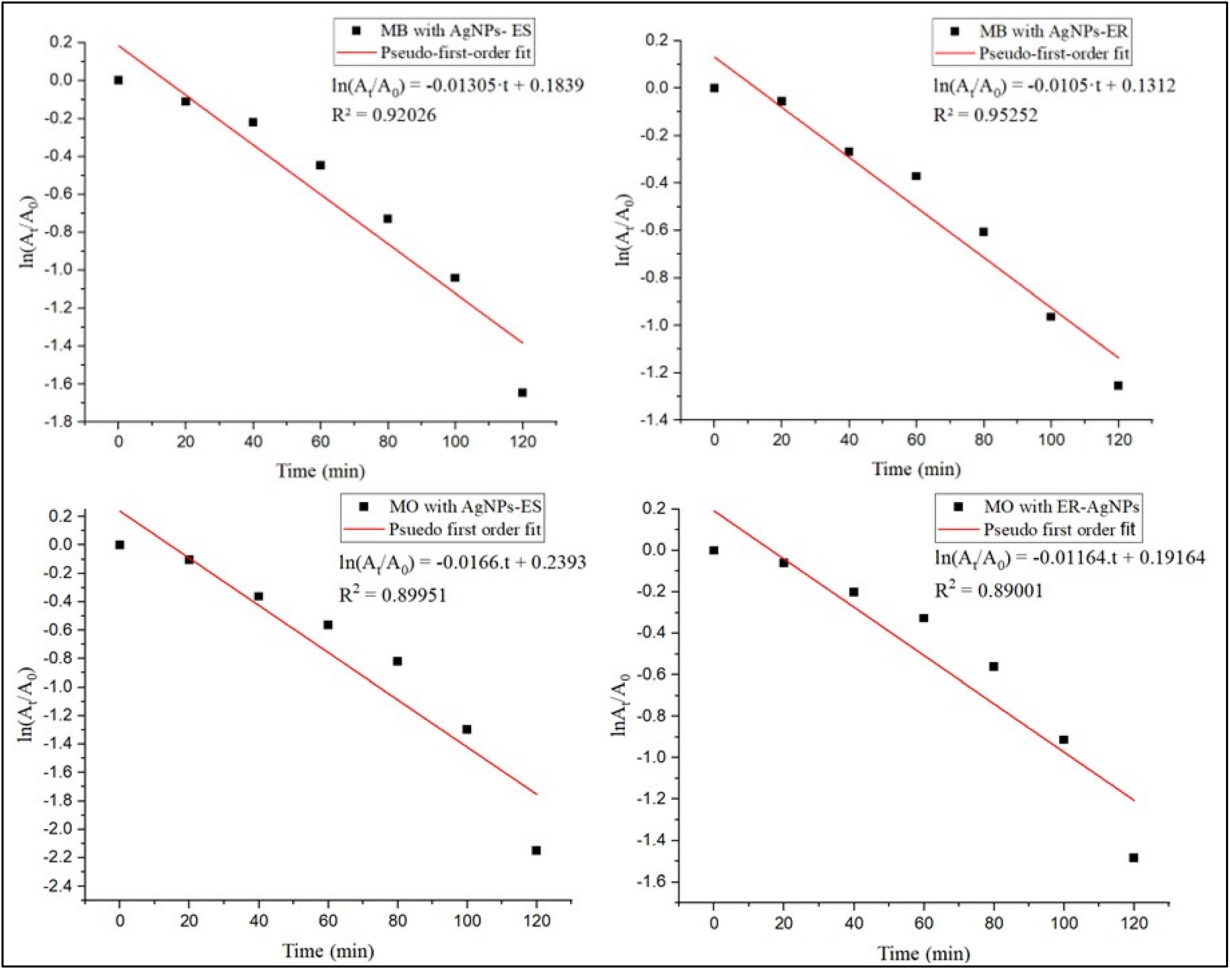
Linear plot of ln (*A*_*t*_/*A*_0_) *vs.* time for degradation of MB and MO dyes with Ag NPs.

All dye-NP systems fit well to the pseudo-first order, as indicated by rather significant regression coefficients (*R*^2^). Linear regression was applied to the plots of ln(*A*_*t*_/*A*_0_) *versus* time (Fig. 15), and the resulting kinetic parameters are summarized in Table 2. MO degradation by Ag NPs-ES had the highest rate constant (*k* = 0.0166 min^−1^), followed by MB with Ag NPs-ES (*k* = 0.01305 min^−1^), MO with Ag NPs-ER (*k* = 0.01164 min^−1^), and MB with ER-Ag NPs (*k* = 0.0105 min^−1^). This tendency suggests that MO degrades at a higher rate than MB, possibly due to its simpler molecular structure and improved affinity to the surface. Ag NPs-ES possess high catalytic performance compared to Ag NPs-ER, which may also be associated with the variations in the surface morphology, active sites, and phytochemical capping agents of the corresponding plant extracts (Table 4).

**Table 2:** Comparative antibacterial activity of plant-based Ag NPs

Plant source	Target bacteria	ZOI (mm)	References
*E. ritro*	*S. aureus*, *B. subtilis*, *B. licheniformis*, and *E. coli*	22.33 ± 0.33, 20.66 ± 0.66, 15.33 ± 0.88, and 15 ± 0.57	Present study
*E. spinosus*	*S. aureus*, *B. subtilis*, *B. licheniformis*, and *E. coli*	24.66 ± 1.45, 24 ± 1, 21.66 ± 0.88, and 21 ± 0.57	Present study
*Echinops* sp	*S. aureus*, *E. coli*, *P. aeruginosa*, and *E. aerogenes*	18, 14, 16, and 15	37
*E. ritro*	*Escherichia coli*, *Enterococcus faecalis*, *Proteus mirabilis*, *Enterococcus hirae*, and *Bacillus cereus*	33 ± 12.5, 20 ± 0.00, 27 ± 6.50, and 30 ± 0.00, 19 ± 0.00	35
*Serratia nematodiphila*	*B. subtilis*, *K. planticola* and *P. aeruginosa*	17 ± 0.57, 14 ± 0.54, and 18 ± 0.33	38
*Psidium guajava*	*B. aryabhattai*, *A. creatinolyticus*, *B. megaterium*, *B. subtilis*, *E. coli*, and *A. faecalis*	20.71 ± 0.23, 22.45 ± 0.61, 21.36 ± 0.28, 19.96 ± 0.45, 23.71 ± 0.12, and 26.18 ± 0.25	39
*Lysiloma acapulcensis*	*E. coli*, *S. aureus*, *P. aeruginosa*, and *C. albicans*	18.0 ± 1.3, 16.0 ± 1.0, 15.0 ± 0.5, and 19 ± 0.5	40
*Bergenia ciliata*	*S. aureus*, *Bordetella bronchiseptica*, and *E. aerogens*	8.5 ± 1.0, 8 ± 0.521, and 11 ± 1.52	41
*Elettaria Cardamomom*	*B. subtilis* and *Klebsiella planticola*	12 and 12	42

The results are consistent with literature that showed a diverse level of degradation when using plant-derived Ag NPs depending on the extract composition, NPs size, and surface chemistry (Table 3).

**Table 3:** Degradation efficiency (%) of dyes using Ag NPs (Ag NPs) synthesized from various plant sources

Plant source	Dye	Degradation (%)	Reference
*E. spinosus*	MB and MO	80% and 88%	This work
*E. ritro*	MB and MO	71.2 and 74.8%	This work
*Terminalia arjuna*	MO, MB, Congo red (CR), and 4-4-nitrophenol (4-NP)	86.68% (MO), 93.60% (MB), 92.20% (CR), and 88.80% (4NP)	55
*Zingiber officinale*	MB	99.9%	56
*Juglans Regia* L	Industrial effluent dye	82%	57
*Crataegus pentagyna*	Rhodamine B (RhB), eosin (EY), and (MB)	85% (RhB), 70% (EY) and 78% (MB)	58
*Sanvitalia procumbens*	Orange G and direct blue-15	70.61% (orange G) and 70% (direct blue-15)	59
*Brassica oleracea* var. *botrytis*	MB	97.57%	60
*Caralluma acutangular*	CR and MB	95.24% (CR) and 96.72% (MB)	61
*Chlorella vulgaris*	MB	96.51%	62
*Antidesma acidum*	MB and CR	81% (MB) and 90% (CR)	63
*Vitex trifolia*	MB	95%	64

**Table 4:** Kinetic parameters for dye degradation by Ag NPs

Dye	NPs	Rate constant (*k*) (min^−1^)	R^2^ value
MB	ES-Ag NPs	0.01305	0.92026
MB	ER-Ag NPs	0.0105	0.95252
MO	ES-Ag NPs	0.0166	0.89951
MO	ER-Ag NPs	0.01164	0.89001

**Fig. 16:**
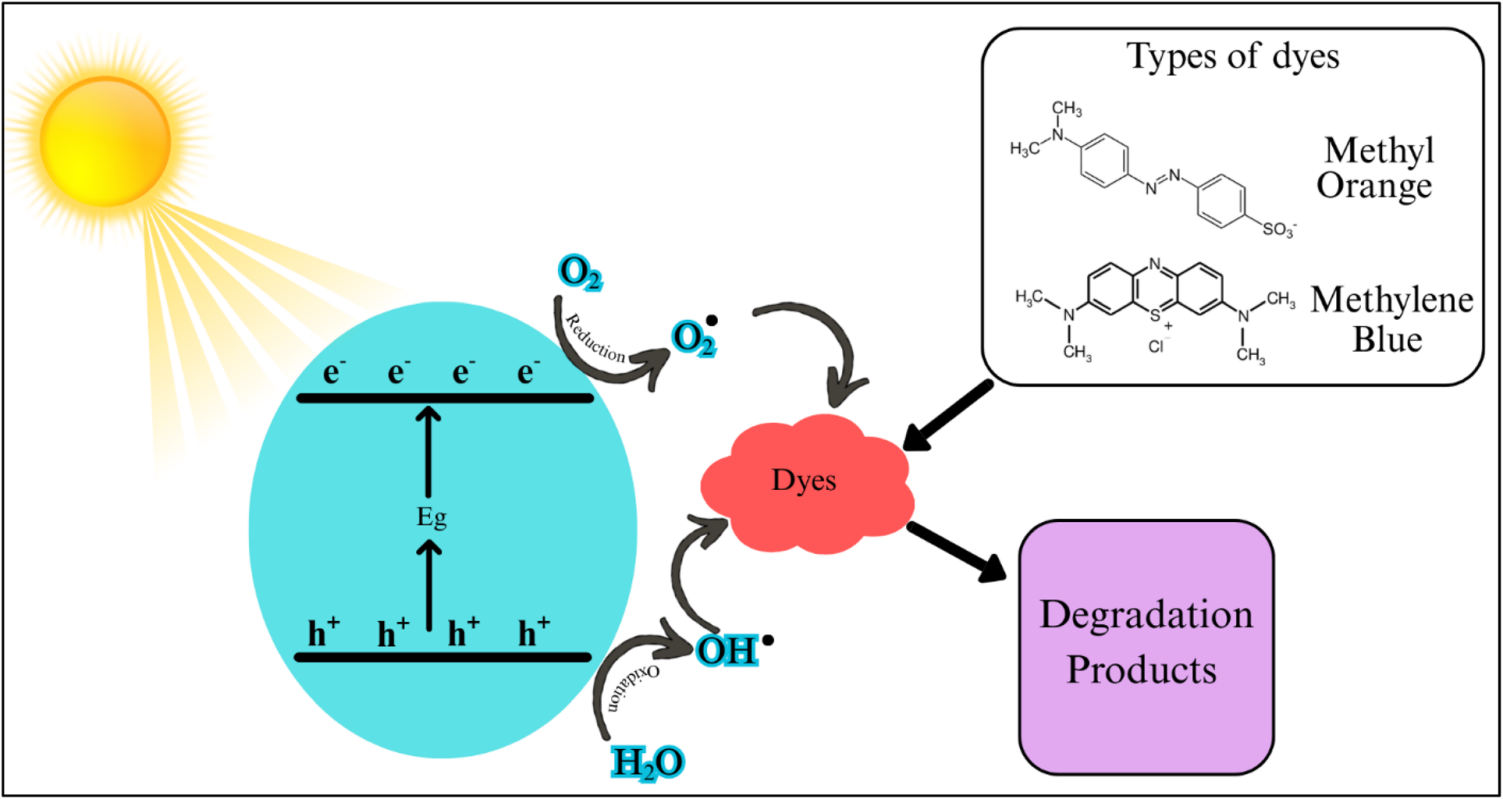
Plausible mechanism of dye degradation by Ag NPs.

The photocatalytic degradation of MB and MO by biosynthesized Ag NPs can be described as a surface plasmon resonance (SPR)-mediated mechanism under sunlight. When Ag NPs are exposed to solar light, the conduction band electrons of the particle collectively oscillate to create hot (energetic) electrons and holes. These charge carriers move to the nanoparticle surface and undergo redox reactions. The excited electrons (e^−^) react with the oxygen molecules (dissolved) to produce the superoxide radicals, whereas the holes (h^+^) generated by the photogeneration react with water or hydroxide ion to produce hydroxyl radicals (˙^−^OH).^65^ These highly reactive oxygen species (ROS) serve as the main oxidizing agent toward the cleavage of chromophoric structures of the dye molecules in these instances, the azo (–N=N–) linkage is targeted by the ROS, resulting in quick cleavage of the bond and the formation of low-molecular-weight aromatic intermediates, which are further mineralized to CO_2_, H_2_O, and inorganic ions. In the case of MB, the process of degradation occurs by oxidative ring-opening of the heterocyclic aromatic structure and subsequently by demethylation and mineralization (Fig. 16).^66,67^

The increased degradation efficiency of Ag NPs-ES can be explained by the enhanced surface morphology, reduced agglomeration, and successful phytochemical capping that increase charge separation, ROS generation, and surface adsorption of the dye molecules.

#### 3.5.6. Reusability and stability

The reusability and kinetic stability of the biosynthesized silver nanoparticles were measured by the successive cycles of photocatalytic degradation of methylene blue (MB) and methyl orange (MO) under the same experimental conditions. The Ag NPs were centrifugally collected after every single cycle and then washed completely with distilled water, followed by drying and reused in the next run.

In the case of MB degradation, Ag NPs-ES displayed a slow loss of photocatalytic efficiency to 79.3, 78.4, 76.3, and 74.0 after the 2nd, 3rd, 4th, and 5th cycles, respectively. Comparatively, Ag NPs-ER exhibited a stronger decrease, with efficiencies of 71.2, 69.0, 67.3, and 63.2 across the same cycles.

In the same way, Ag NPs-ES retained better catalytic activity during MO degradation, with degradation efficiencies of 88.0, 87.1, 85.9, and 85.0 in the second to the fifth cycles, respectively. On the other hand, Ag NPs-ER had lower stability, and the efficiencies reduced to 74.0, 72.2, 71.0, and 68.8 in repeat usage. The observed decrease in the photocatalytic activity with the consecutive cycles could be explained by the fact that the surface is partly contaminated, the nanoparticles are somehow agglomerated, or the active sites are partially blocked by the reaction intermediates. However, the relatively enhanced stability of Ag NPs-ES can probably be related to the better phytochemical capping obtained with the help of *Echinops spinosus*, promoting the surface protection and preventing the agglomeration. In conclusion, the results show that the produced Ag NPs, especially Ag NPs-ES, have excellent reusability and are structurally stable, which underscores the possibility of practical application in treating wastewater.

#### 3.5.7. Long-term stability and environmental conditions

Photocatalysts can be used in practice only when their stability under the conditions of the real environment is guaranteed. In the current research, Ag NPs biosynthesis was conducted in near-neutral conditions (pH ≈ 6) with no external temperature or thermal treatment added, which means that the synthesis process itself does not cause any thermal stress on the nanoparticles and is, therefore, environmentally harmless.

The fact that the photocatalytic behaviour of both MB and MO has been preserved through the course of five consecutive degradation cycles indicates that the Ag NPs synthesized in this paper have a stable structure even in near-neutral aqueous environments. The fact that efficiency was not lost suddenly also suggests that there was a small agglomeration or dissolution during repeated use of the photocatalyst. Also, FTIR analysis establishes the existence of phytochemical capping agents from *Echinops* species, which promote colloidal stability by offering steric hindrance and protecting the nanoparticle surface against oxidation or aggregation.

Although longer storage stability and systematic stress testing in terms of pH, temperature were not evaluated in this experiment, the near-neutral synthesis conditions, multi-cycle reusability, and sustained photocatalytic performance of the Ag NPs show positive stability under operational conditions. Future research will be done on long-term storage analysis and stability analysis within wider pH and temperature conditions to prove practical applicability further.

## 4. Conclusion and future directions

Silver nanoparticles (Ag NPs) of *Echinops ritro* (Ag NPs-ER) and *Echinops spinosus* (Ag NPs-ES) were produced using a green, eco-friendly process. The nanoparticles exhibited strong antibacterial activity against the target bacterial strains and efficiently degraded methylene blue and methyl orange dyes. At the mechanistic scale, the photocatalytic effect may be credited with the creation of reactive oxygen species on the surfaces of Ag NP under the light of the sun, which oxidizes the dye molecules. The reduced size and large surface area increase the interaction between the nanoparticles and the bacterial cells. Adsorption–desorption studies were not quantitatively assessed and will be explored in future work to further distinguish adsorption from photocatalytic effects.

The green synthesis of Ag NPs by means of *Echinops ritro* (Ag NPs-ER) and *Echinops spinosus* (Ag NPs-ES) is ecological, economical, and easily reproducible in comparison with conventional chemical techniques. The nanoparticles are highly stable, well-morphed, and have high antibacterial and photocatalytic activity, which proves a new and feasible methodology with the prospect of environmental and biomedical uses. Ag NPs-ES showed better antibacterial and photocatalytic characteristics than Ag NPs-ER, which were among the synthesized nanoparticles. This increased activity could be explained by the increased concentration of reducing and capping phytochemicals in the *Echinops spinosus* extract (ES), which led to a decrease in particle size, an increase in surface area, and the subsequent enhancement of reactivity and interaction of the nanoparticles with bacterial cells and the dye molecules.

As a future direction, the chronic cytotoxicity and biocompatibility of these Ag NPs against mammalian cell models should be examined first before moving to clinical or biomedical uses. Synergistic research may be carried out using synergistic combinations of Ag NPs and conventional antibiotics to identify whether Ag NPs potentiate antibacterial activity. Further research into photocatalytic efficiency under different light exposures and dye concentrations, and in contact with real wastewater samples, would allow transferring laboratory-scale results to real-world practice. Also, a chemical characterization of *Echinops* species at the phytochemical level has the potential to determine the identity of active compounds involved in reductions and stabilization, thereby allowing optimization of the process to regulate the size of NPs and automation of the functional properties. Further research will involve fine-scale colloidal stability investigations, which comprise: the evaluation of the size distribution using DLS, the evaluation of zeta potentials, and a thorough surface chemistry analysis to further confirm the physicochemical character of the synthesized Ag nanoparticles.

## Conflicts of interest

All the authors declare that they have no conflicts of interest regarding the publication of this manuscript.

## Funding

This work was supported financially by the Deanship of Scientific Research at King Faisal University, under Ambient Researcher [Grant, KFU260442].

## Data Availability

All the data supporting the findings of this study are provided in the manuscript.
